# Nosocomial Rotavirus Gastroenteritis in pediatric patients: a multi-center prospective cohort study

**DOI:** 10.1186/1471-2334-10-235

**Published:** 2010-08-09

**Authors:** Filippo Festini, Priscilla Cocchi, Daniela Mambretti, Bruna Tagliabue, Milena Carotti, Daniele Ciofi, Klaus P Biermann, Roberto Schiatti, Franco M Ruggeri, Fernando Maria De Benedictis, Alessandro Plebani, Alfredo Guarino, Maurizio de Martino

**Affiliations:** 1 Department of Pediatrics, University of Florence, Florence, Italy; 2 Department of Pediatrics, University of Naples "Federico II", Naples, Italy; 3 Department of Pediatrics, University of Brescia, Brescia, Italy; 4 Department of Pediatrics, "Salesi" Children's Hospital, Ancona, Italy; 5 Infection Control Unit, "Meyer" Children's Hospital, Florence, Italy; 6 Department of Veterinary Public Health & Food Safety, National Institute of Health-Istituto Superire di Sanità, Rome, Italy

## Abstract

**Background:**

Few data are available on the incidence of nosocomial Rotavirus infections (NRVI) in pediatric hospitals and on their economic impact. The goals of this study were: to evaluate the incidence of NRVI in various Italian pediatric wards during the course of two peak RV seasons; to investigate possible risk factors for NRVI; to estimate the costs caused by NRVI.

**Methods:**

prospective cohort study. Population: all the children under 30 months of age who were admitted without any symptom or diagnosis of gastroenteritis in the pediatric hospitals of Florence, Naples, Brescia and Ancona, Italy, during the winter-spring periods 2006-2007 and 2007-2008. Serial RV rapid tests and clinical monitoring were carried out on the cohort. Telephone interviews were performed from 3 to 5 days after discharge.

**Results:**

520 out of 608 children completed the study (85.6%). The overall incidence of NRVI was 5.3% (CI95% 3.6-7.5), (7.9 per 1,000 days of hospital stay, CI 95% 5.3-11.3). The average duration of hospital stay was significantly longer for children who had NRVI (8.1 days, SD 5.4) than for non-infected children (6.4 days, SD 5.8, difference 1.7 days, p = 0.004). The risk of contracting NRVI increased significantly if the child stayed in hospital more than 5 days, RR = 2.8 (CI95% 1.3-6), p = 0.006. In Italy the costs caused by NRVI can be estimated at 8,019,155.44 Euro per year. 2.7% of the children hospitalized with no gastroenteritis symptoms tested positive for RV.

**Conclusions:**

Our study showed a relevant incidence of NRVI, which can increase the length of the children's stay in hospital. Limiting the number of nosocomial RV infections is important to improve patients' safety as well as to avoid additional health costs.

## Background

Rotavirus (RV) is the most frequent cause of viral gastroenteritis in children under 5 years of age. The virus can cause severe diarrhea and dehydration, especially in children aged 6 to 24 months. In developing countries, acute gastroenteritis due to RV infection (RV AGE) causes the death of approximately 440,000 children every year [1,2]. In the USA, RV is responsible for the hospitalization of 58,000 to 70,000 children every year [3].

The RV genus of the *Reoviridae *family is very diverse, as it consists of different groups (A-G) and of different types based on the characteristics of the surface proteins VP7 (G = glycoprotein) and VP4 (P = protease-sensitive protein). To date, at least 23 G types and 31 P types of group A RVs, the group which most commonly infects humans, have been differentiated [4,5].

The virus is mainly transmitted by feco-oral route or by direct contact, but it can occasionally be transmitted through droplets. Since the virus is stable in the environment, transmission can occur through the ingestion of contaminated water and food, and through contact with contaminated surfaces and objects. Cross-infection through contamination of the hands is probably the most common transmission route in healthcare settings. RV AGE has an incubation period of 1 - 3 days, which is followed by the sudden onset of watery diarrhea, with possible dehydration, vomiting and fever lasting from 4 to 7 days. In the temperate zones of the planet, the virus has seasonal peaks (in the Northern hemisphere from November to March), whereas in tropical regions RV infections occur all year round [6].

In 2007 the World Health Organization recommended the inclusion of rotavirus vaccination in the national immunization programs of countries in which RV gastroenteritis has a substantial public health impact [7]. Evidence of the effectiveness of immunization programs is available for the two existing RV vaccines, both in industrialized and in developing countries [8-10]. Recent data suggest that the RV vaccine coverage is as high as 70% in some USA states [11]. Reported RV vaccine coverage in European countries varies from 35% to 85% [12].

In industrialized countries, RV infection is only rarely a cause of death for children. Nevertheless, the high number of hospitalizations due to RV indicates that the economic and social impact of the disease deserves attention [13].

In particular, RV infections contracted by hospitalized children is a source of worry. In pediatric hospitals, the reservoir of infection is represented by already infected children, while the vehicle can be toys or other objects which are handled by children, also via the hands of mothers and health care workers which have not been washed correctly. The infection contracted in the hospital setting causes the child to stay longer in hospital as well as additional economic and social costs [14]. Asymptomatic RV infections are a particular cause of concern. These infections lack the symptoms of vomiting and/or diarrhea, or infected people often show nonspecific symptoms such as fever, headache, nausea, and fatigue [15]. Recent studies showed relevant prevalences of asymptomatic RV infections both among adults and children [15,16].

At present, the number of studies on the incidence of nosocomial RV infections (NRVI) in pediatric hospitals [16-26] and on their economic impact [14,16,27-29] is limited.

The incidence of NRVI in hospitals may be underestimated and little attention may be given to prevention and to the increase in the social costs of the disease, e.g. longer hospital stay (direct costs), as well as in absences from work of family members who assist the young patient (indirect costs) [30,31].

Additional data on the incidence of NRVI are useful to evaluate the risks linked with healthcare, to plan the use of available resources, and to estimate healthcare costs [32,33].

To calculate the incidence of NRVI with acceptable precision, it is necessary to carry out prospective population studies including all the children hospitalized during a period of reference. Given that the incubation period of RV infection can vary from 24 to 72 hours and in order to be able to detect all NRVI cases, tests on the feces of all the patients hospitalized without gastroenteritis symptoms should be carried out upon admittance, as well as during the period of hospital stay if gastroenteritis symptoms manifest themselves. Furthermore, all children must be clinically monitored for up to 72 hours following discharge from the hospital [16,34].

The primary objective of our study was to evaluate the incidence of NRVI in various pediatric wards throughout Italy during the course of two peak RV infection periods.

The secondary objectives were:

- To investigate possible risk factors for NRVI among the demographic and nosological characteristics of the hospitalized children.

- To estimate the additional health costs caused by nosocomial RV infections in the periods under consideration.

## Methods

### Study design

We conducted a multi-center observational prospective cohort study during two peak RV infection periods (winter-spring 2006-2007, winter-spring 2007-2008). The choice of the period was based on a preliminary study, which showed that approximately 80% of NRVI cases occur during the winter-spring period [35].

The incidence of nosocomial RV infection was investigated by constantly monitoring the clinical conditions of the whole population of children hospitalized in the participating wards during the study period.

In the first period, the study was carried out in three pediatric wards in two hospitals ("Meyer" Children's Hospital of Florence and Pediatric Unit of the University of Naples "Federico II"). In the second period, the study was carried out in five pediatric wards in four hospitals (the ones previously mentioned, as well as the Pediatric Unit of the Children's Hospital of Brescia and the Pediatric Unit of the "Salesi" Hospital of Ancona). The study setting was represented by the wards which normally care for children up to 30 months of age who do not need intensive care.

Subjects were consecutively recruited from all the children under 30 months of age who were admitted to the participating wards without a diagnosis of RV AGE and without any symptom suggesting gastroenteritis (diarrhea or vomiting with or without fever) in the periods from 24/1/2007 to 31/5/2007 and from 14/12/2007 to 31/5/2008. Informed consent to participation in the study was given by the children's parents. Recruitment was performed upon hospital admission. Upon recruitment of each child in the participating ward, medical history data were obtained.

A rapid RV test was carried out on the first feces emitted by all the recruited subjects after hospital admittance, regardless of their clinical condition.

The children for whom it was not possible to obtain a fecal sample within 24 hours of hospital admission were considered "lost to follow-up". In this case, evidence that the subject was negative for RV upon enrollment and thus susceptible to infection was unavailable.

The children who tested positive for RV upon admittance were considered not recruitable *ex-post*, as they did not meet one of the inclusion criteria.

Each of the enrolled children was visited by a doctor or nurse (including physical examination) at least once a day for the whole duration of their stay in hospital. The pediatricians and nurses performed a clinical evaluation and routine check-up on the children following the indications of the protocol adopted in the ward. When signs and symptoms of gastroenteritis arose during hospital stay, a RV test of the child's feces was carried out.

The rapid RV test was performed again at the moment of discharge from the hospital, irrespective of the child's clinical condition.

Furthermore, the family was contacted via telephone between the 3^rd ^and the 5^th ^day following discharge to answer a number of questions regarding the appearance of signs and symptoms of gastroenteritis after leaving the hospital ward.

The RV test was conducted using a rapid test for RV (Vikia Rota-Adeno Biomerieux, Lyon, France). This test has a 99% sensitivity rate and 100% specificity.

The samples which tested positive for RV were preserved at -20°C and later sent to the Istituto Superiore di Sanità (National Institute of Health, Rome, Italy) for genotyping.

The recruited subjects who tested negative for RV at hospital admittance with the rapid test were considered cases of nosocomial RV infection when the rapid test indicated RV infection at least 72 hours after hospital admittance.

Children were considered lost to follow-up in case of withdrawal of consent by the parents, in case of inability to obtain a fecal sample for RV control upon discharge from the hospital, and in case of inability to carry out a telephone interview within 3 to 5 days after discharge.

The study was conducted in accordance with the latest amended version of the Declaration of Helsinki, with the Good Clinical Practice guidelines and with the European guidelines on conducting clinical studies in pediatrics (CPMP/ICH/99). Authorization was requested and obtained from the ethics committee of all the four hospitals involved in the study.

### Data Analysis

In order to calculate the incidence of NRVI, the numerator is represented by the detected NRVI cases (as previously defined); the denominator is represented by the number of recruited subjects minus the ones lost to follow up.

Afterward, we calculated the incidence per 1,000 days of hospital stay. In this case, the numerator is represented by the number of detected NRVI cases (as previously defined); the denominator is represented by the number of total days spent in hospital by the recruited patients, minus the number of days of stay of the patients lost to follow-up and of the ones with RV infection after admission.

The 95% confidence interval was calculated on the incidence [36].

In order to explore any possible differences in the risk of contracting NRVI based on the various characteristics of the subjects, we carried out an exploratory statistical analysis by stratifying the subjects on the basis of several qualitative and quantitative variables.

The statistical analysis of the differences in the risk of contracting NRVI for dichotomous variables (sex and age range) was conducted by calculating the relative risks and by determining the corresponding 95% confidence interval.

We also studied the difference in age and in the length of hospital stay of children with and without NRVI. The statistical analysis was carried out by comparing the mean age using ANOVA and the length of hospital stay with a non-parametric test (Mann-Whitney).

In order to estimate the additional costs attributable to NRVI, we first estimated the number of children affected by NRVI in Italy in one year: this was done by using the data concerning the hospitalization of children provided by the Istituto Centrale di Statistica Italiano (Italian Central Institute of Statistics) [37,38] and by applying the incidence detected in our study to these data.

For each additional day of hospital stay due to NRVI, the following were calculated:

- the cost of the days spent in hospital, based on the Diagnosis Related Groups [39-41] of the Italian Ministry of Health;

- the cost of lost productivity due to absence from work of one of the parents, on the basis of the data provided by the Italian Central Bank [42,43].

For each of the two study periods, the coordinating center performed three data quality assessments by analyzing all of the clinical assessment forms and by verifying data consistency. Random assessment of data recording quality was also conducted.

### Genotyping

The clinical RV isolates were shipped in dry ice to the National Institute of Health of Rome for strain characterization. A 10% suspension in water was prepared for further rotavirus-specific RT-PCR and nested PCR tests. Nucleic acid extraction and G/P rotavirus typing: double-stranded viral RNA was extracted from 140 ul of the 10% fecal suspension by using a commercial kit (QIAamp viral RNA minikit; QIAGEN) and following the manufacturer's instructions. The RNA was eluted in 50 ul of RNase-free water and stored at -80°C. G and P rotavirus genotyping was performed by using RT-PCR methods, as reported previously [44]. In order to identify the G type, we used the VP7-F and VP7-R consensus primers [45] in RT-PCR. Subsequently, the VP7-R primer was used in a nested multiplex PCR together with G1, G2, G3, G4, G8, G9, and G10 type-specific primers [44-46]. The samples which tested negative by G typing with the primers described above were also tested by using a PCR with G12-specific primers [47]. For P types, we used Con2-Con3 consensus primers [48] in RT-PCR, followed by the standard multiplex PCR including the Con3, in combination with the typing primers P4, P6, P8, P9, P10, and P11 [46,48,49]. VP6 RT-PCR was performed as described previously [50].

## Results

We were able to recruit 608 subjects who met the inclusion criteria.

Out of the 625 subjects who initially met the inclusion criteria, 17 (2.7%) tested positive for RV on the rapid test conducted upon hospital admittance, even without symptoms of AGE. 88 subjects were lost to follow-up (equal to 14.4%), 64 of whom due to the inability to take a stool sample within 24 hours of admittance and another 24 due to the inability to perform a final evaluation (faeces exam or telephone interview).

**Figure 1:**
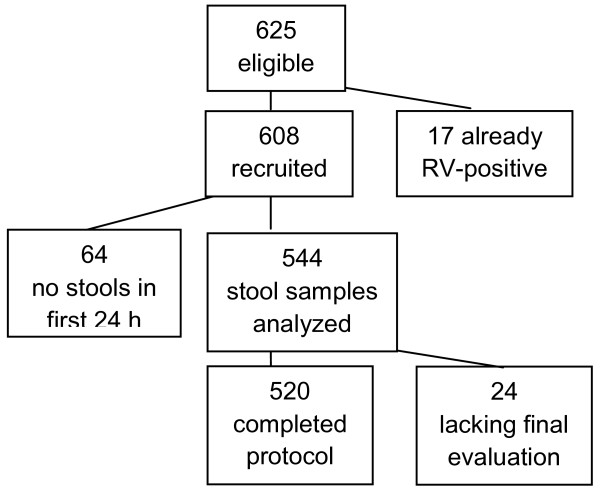
**Flowchart of the subjects included in the study**.

Figure 1 shows the flowchart of the subjects included in the study.

The 520 children who completed the study had a mean age of 9.4 months (SD 8.2) and stayed in hospital for an average of 6.5 days (SD 5.8). 41.3% of them were female. With respect to age, 11.3% of children were newborns, 34.8% were aged from 1 to 6 months, 19.2% from 7 to 12 months and 34.7% from 13 to 30 months.

### Detection of NRVI

During the study period, 28 cases of NRVI were detected. Sixteen NRVIs out of 229 subjects were detected in the first season and 12 out of 291 in the second season.

The total incidence of NRVI was 5.3% (CI95% 3.6-7.5), 6.9% in the first season (CI95% 4.1-10.8) and 4.1% in the second season (CI95% 2.2-6.9).

The incidence per day of hospital stay was also calculated. The overall incidence of NRVI (per 1,000 days of hospital stay) was 7.9‰ (CI95% 5.3-11.3): 11.5‰ in the first season (CI95%6.6-18.7) and 6.1‰ in the second season (3.2-10.8).

Based on the telephone interviews performed from 3 to 5 days after hospital discharge, 29 additional cases of suspected gastroenteritis emerged after the subjects returned home and were thus associated with being hospitalized. However, it was not possible to obtain diagnostic confirmation of these cases with an RV test on stools.

Table 1 shows the distribution of the reasons of hospitalization among the 28 children who had NRVI.

**Table 1:** Distribution of the reasons of hospitalization among the 28 children who had NRVI.

**reason of admission**	**n**	**%**	**reason of admission**	**n**	**%**
	
pneumonia	8	28.6	gastro-oesophageal reflux	1	3.6
	
bronchiolitis	1	3.6	pyelonefritis	2	7.1
	
acute problems in chronic patients	1	3.6	failure to thrive	1	3.6
	
febrile seizure	2	7.1	trauma	1	3.6
	
anemia	3	10.7	other	7	25.0
	
non febrile seizure	1	3.6			

### Statistical analysis

The risk of contracting NRVI for females was half the one for males (3.7% vs. 6.5%). However, the difference is not statistically significant (RR = 0.57, CI 0.25-1.27).

With respect to age, the incidence of NRVI was 4.23 (CI 95% 2.3-7.6) in children under 6 months of age (n = 236). In children aged 6 months or more (n = 284), the incidence of NRVI was 6.33 (CI 95% 4-9.7), RR = 1.49, CI 95% 0.7-3.1.

Children in their 9^th ^month of age (n = 19) turned out to be at a higher risk of contracting NRVI (21.1%) than children of all other ages, RR 4.39, CI95% 1.69-11.4.

No statistically significant difference was found between the mean ages of children with and without NRVI (9.3 months and 9.4 months respectively, p = ns).

There was no statistically significant difference also between the mean ages of children with and without NRVI stratified by sex and season of study (1^st ^or 2^nd ^season of the study).

Mean hospital stay resulted significantly longer (8.1 days, SD 5.4) for children who had NRVI (n = 28) than for the ones who did not contract it (6.4 days, SD 5.8, difference of 1.7 days, p = 0.004) (n = 492). On average, hospital stay was thus 26.5% longer for children who had contracted the nosocomial infection.

Table 2 shows the difference between the mean length of hospital stay for children with and without NRVI stratified by sex and season.

**Table 2:** Differences between the mean length of hospital stay for children with and without NRVI stratified by sex and season

	Children with NRVI	Children without NRVI	p
Males	7.6 (sd 7.2) n = 20	6.2 (sd 8.3) n = 286	0.03

Females	9.5 (sd 5.4) n = 8	6.7 (sd 6.1) n = 206	0.03

1st season	7 (sd 2.6) n = 16	6.1 (sd 5.9) n = 213	0.01

2nd season	9.6 (sd 7.6) n = 12	6.6 (sd 5.7) n = 279	ns

Among children whose hospital stay was longer than 5 days (n = 225) there were 19 cases of NRVI vs 9 cases among children with an hospital stay up to 5 day long (n = 295), with a RR = 2.8 (CI95% 1.3-6), p = 0.006.

Among children admitted to hospital in the month of March (n = 131) there were 12 cases of INRV vs 16 cases among children admitted in all other months (n = 389), with a RR of 2.2 (CI95%1.08-4.5), p = 0.02.

### Cost analysis

In Italy approximately 885,000 children aged 0 to 14 require hospital admission every year. Approximately 414,000 of them are under 30 months of age, 36.9% of whom are hospitalized during the period of reference of our study (December-May) [37,38]. Therefore, the total number of children under 30 months of age who are hospitalized during the RV peak period is approximately 152,766.

Based on the incidence found in our study, one could estimate that in Italy approximately 8,097 children under 30 months of age hospitalized in the December-March period contract NRVI every year.

Based on the differences we found in our study in the length of hospital stay between the children affected and the ones not affected by NRVI, the total increase in hospital stay caused by NRVI can be estimated to be 13,764.9 days. The average cost of a day in hospital in an Italian pediatric ward is 434.58 Euro [39-41], while the average unitary cost of a lost day of work is 148.00 Euro [42,43].

At a national level, the additional health costs derived from longer hospital stay due to NRVI can thus be estimated to be 8,019,155.44 Euro per year.

The cost of one RV vaccination is 164 Euro. Therefore, vaccinating all the subjects included in our study would have cost 85,280 Euro. Based on the differences we found in the length of hospital stay between the children affected and the ones not affected by NRVI, we can speculate that through RV vaccination, the total number of days spent by children with NRVI in hospital (229 days) would have been reduced to 168.3. Saving 60.7 hospital days corresponds to an estimated reduction in healthcare expenditure of 35,362.60 Euro per year.

### Typing of collected isolates

The results of genotyping show that in winter the G1 P[8] genotype is more common (80%, n = 12). Other genotypes found during the winter season were G9 P[8] (13.3%, n = 2) and G3 P[8] (6.6%, n = 1).

The distribution of the genotypes responsible for the contraction of NRVI in spring is instead more varied: G1 P[8] (50%, n = 6), G9 P[8] (41.6%, n = 5), G2 P[4] (8.3%, n = 1). In one case, it was not possible to type the isolate.

## Discussion

At present, several retrospective studies providing estimates of the incidence of NRVI in children are available. Compared to them, prospective cohort studies can provide more reliable and less biased data. However, to date only few researches on the incidence of NRVI have been conducted using a prospective cohort study design and the existing ones were mainly carried out on small populations [19-26].

Our results give an updated and representative picture of the epidemiology of nosocomial RV infections, which affect more than 5% of the children under 30 months of age hospitalized in winter and in spring. This also confirms the clinical and social relevance of this type of infection. The total incidence of NRVI detected in our study deserves attention and it is consistent with the results of other studies having the same design [19-26]. A 2006 review about NRVI incidence across Europe reports that NRVIs account for 0.3-27.7% of all hospital admissions and for 1.6-15.8 per 1,000 days of hospital stay [14].

A limit of our study could be the fact that the observed cohort had a loss to follow-up of 14.4%, a statistically acceptable percentage which should not to be overlooked, but which does not alter the reliability of results.

Another possible limit of our study may be the fact that no information is available about the percentage of recruited children immunized against RV and about the percentage of breast-fed babies among them. This information could have given important indications on how to evaluate the incidence of NRVI resulting from our study. Breastfeeding is indeed associated with a reduced incidence of gastroenteritis [51]. Our data show a reduced incidence of NRVI in children under 6 months of age, which might be explained both by breastfeeding and by the presence in younger infants of transplacentally transferred maternal anti-RV antibodies [52]. However, the difference is not statistically significant.

With respect to the possible risk factors for NRVI, the mean age of the children affected by NRVI did not differ from the one of children who were not. No significant difference in the incidence of NRVI in the two genders was found.

In line with the data of other studies [14], the period of highest risk for NRVI turned out to be the month of March. Our study also highlighted that the length of hospital stay influences the risk of contracting NRVI. In fact, the risk increases in a statistically significant manner after the fifth day in hospital.

Our study also showed that NRVI brings about a statistically significant and clinically relevant increase in hospitalizations, thus causing higher, non-negligible costs for the healthcare and social systems, also in relation to the total healthcare costs of a country.

Our data do not seem to suggest a possible decrease in the direct costs of hospitalization following RV vaccination. However, our study population is limited and additional research is necessary to evaluate the possible impact of RV vaccination on the additional costs highlighted in our study.

With respect to typing isolates responsible for NRVI, recent global epidemiology studies, used to monitor the emergence of new strains, highlighted that 88% of the strains which can infect human beings have G1, G2, G3 and G4 genotype in combination with P[8] or P[4] elements [53]. The results of our study are consistent with these previous findings.

It is worth noting that the genotypes of the isolates found in our study are all covered by the two existing Rotavirus vaccines (Rotateq, Sanofi Pasteur MSD, Lyon and Rotarix, GlaxoSmithKline Biologicals, Rixensart)

Since the transmission of RV occurs primarily by contact, the results of our study confirm the importance of insisting on handwashing, on the implementation of appropriate hygiene standards and on prevention, as recommended also in the existing guidelines [54-56] for relatives of hospitalized children and for hospital staff.

It is noteworthy that 2.7% of the recruitable subjects who were hospitalized for reasons other than gastroenteritis, and who had no gastroenteritis symptoms, tested positive for RV. These children can become a source of outbreaks of NRVI within the ward where they are admitted. Using the RV rapid test upon hospital admission during the period of higher risk of NRVI for children under 30 months of age might be a way to reduce the impact of NRVI in pediatric hospitals. However, additional studies are needed to confirm this hypothesis.

## Conclusion

Nosocomial Rotavirus infection is an important problem from a clinical, organizational and economic point of view. Although RV is a marginal cause of death in Western countries, limiting the number of nosocomial RV infections is important both to improve patients' safety and to avoid additional health care costs.

## Competing interests

This research was entirely funded by Sanofi Pasteur.

The author declares that no other financial or non financial competing interest exists.

## Authors' contributions

All authors read and approved the final manuscript. FF conceived the study, participated in its design and coordinated it, carried out the analysis and interpretation of data and prepared the manuscript, PC coordinated the acquisition of data and performed the RV rapid tests, DM coordinated the recruitment and acquisition of samples in center 2, BT coordinated the recruitment and acquisition of samples in center 3, MC coordinated the recruitment and acquisition of samples in center 4, DC participated in the design and analysis of data, KPB coordinated the recruitment and acquisition of samples in center 1, RS participated in the interpretation of data, FMR Carried out the genotyping of RV, FMDB participated in the recruitment and acquisition of samples in center 4, AP participated in the recruitment and acquisition of samples in center 3, AG participated in the recruitment and acquisition of samples in center 2, MDM Participated in the design of the study and interpretation of data.

## Pre-publication history

The pre-publication history for this paper can be accessed here:

http://www.biomedcentral.com/1471-2334/10/235/prepub
